# Neutrality, Cross-Immunity and Subtype Dominance in Avian Influenza Viruses

**DOI:** 10.1371/journal.pone.0088817

**Published:** 2014-02-25

**Authors:** Vicki L. Brown, John M. Drake, Heather D. Barton, David E. Stallknecht, Justin D. Brown, Pejman Rohani

**Affiliations:** 1 Department of Ecology & Evolutionary Biology, University of Michigan, Ann Arbor, Michigan, United States of America; 2 Center for the Study of Complex Systems, University of Michigan, Ann Arbor, Michigan, United States of America; 3 Odum School of Ecology, University of Georgia, Athens, Georgia, United States of America; 4 Southeastern Cooperative Wildlife Disease Study, University of Georgia, Athens, Georgia, United States of America; 5 Fogarty International Center, National Institutes of Health, Bethesda, Maryland, United States of America; University of Oxford, Viet Nam

## Abstract

Avian influenza viruses (AIVs) are considered a threat for their potential to seed human influenza pandemics. Despite their acknowledged importance, there are significant unknowns regarding AIV transmission dynamics in their natural hosts, wild birds. Of particular interest is the difference in subtype dynamics between human and bird populations–in human populations, typically only two or three subtypes cocirculate, while avian populations are capable of simultaneously hosting a multitude of subtypes. One species in particular–ruddy turnstones (*Arenaria interpres*)–has been found to harbour a very wide range of AIV subtypes, which could make them a key player in the spread of new subtypes in wild bird populations. Very little is known about the mechanisms that drive subtype dynamics in this species, and here we address this gap in our knowledge. Taking advantage of two independent sources of data collected from ruddy turnstones in Delaware Bay, USA, we examine patterns of subtype diversity and dominance at this site. We compare these patterns to those produced by a stochastic, multi-strain transmission model to investigate possible mechanisms that are parsimonious with the observed subtype dynamics. We find, in agreement with earlier experimental work, that subtype differences are unnecessary to replicate the observed dynamics, and that neutrality alone is sufficient. We also evaluate the role of subtype cross-immunity and find that it is not necessary to generate patterns consistent with observations. This work offers new insights into the mechanisms behind subtype diversity and dominance in a species that has the potential to be a key player in AIV dynamics in wild bird populations.

## Introduction

Avian influenza viruses (AIVs) have long been a source of concern because of their potential to cause a human influenza pandemic. Indeed, every influenza virus implicated in human pandemics in history has contained gene segments of avian origin [1]–[4]. The current threats from avian influenza are thought to come from H5N1, which has devastated poultry across Asia [5] and H7N9, the human emergence of which, surprisingly, was not preceded by mass die-offs in poultry or wild birds [6]. Given that wild birds represent the natural reservoir for influenza A viruses, responsible for the generation and maintenance of genetic diversity, understanding the population biology of avian influenza viruses is important.

While subtype diversity in human seasonal influenza viruses is limited to H3N2 and H1N1 [7], field sampling of wild bird populations has identified numerous coexisting viral subtypes [8]–[10]. Surprisingly, the mechanisms underpinning the community ecology of AIV subtypes remain poorly understood. Identifying the factors that determine coexistence in multi-pathogen systems is an interesting scientific question in its own right [11]–[15], but in the case of influenza viruses it also has applied significance given their potential for cross-species transmission and ultimately infection in humans [1].

Numerous studies of avian influenza viruses have focused on prevalence and transmission dynamics in ducks and gulls, leading to the observation that each supports a wide range of somewhat distinct virus subtypes [10]. In comparison, less is known about the role of other species in the maintenance of AIV subtype diversity in the wild. In particular, shorebirds are known to harbour a wide range of subtypes, including viruses that are typically found in either duck or gull reservoirs [10], though the mechanisms that allow for this are not known. One location where AIV dynamics in shorebirds and in ruddy turnstones - *Arenaria interpres* - in particular has been extensively studied is Delaware Bay, where these birds routinely exhibit high prevalence levels during their spring migration [9], [16], [17]. This species may be critical in the maintenance and geographic spread of AIVs through North American wild bird populations, as they are both competent hosts for several AIV subtypes and long-distance migrants.

Previous research has attempted to identify the key elements that make Delaware Bay an AIV ‘hotspot’ [16], especially the impact of multiple interacting bird species and, crucially, seasonality in migration, breeding, mortality and transmission [17], [18]. However, this work did not address subtype diversity, coexistence and frequency. In this paper, we attempted to explore the possible explanations for the coexistence of multiple subtypes in ruddy turnstones by first presenting and analysing two sources of subtype-specific AIV data from ruddy turnstones in Delaware Bay. Using these data, we questioned examined patterns of subtype dominance and diversity, as these signatures can provide insight into the subtype interactions taking place within the species. We identified both random and non-random patterns, so we adopted a mechanistic multi-subtype transmission model that is neutral (i.e. does not assume subtype differences) and asked whether such a model is capable of capturing the dominance and diversity patterns observed in the data. Our model is an extension of that presented in [17]; it comprises a stochastic, multi-host, multi-subtype system that incorporates the key seasonal elements mentioned above.

To establish whether cross-immunity or transmission route played a role in the simulated dominance or diversity patterns, we systematically varied these parameters, while always maintaining identical subtype dynamics. We calculated Simpson’s diversity index, change in rank vs. rank (of subtype) and rank-abundance for both the model and field data and compared the results, finding that, for certain parameter sets, a neutral model incorporating demographic stochasticity was capable of creating patterns similar to those observed in the data. We followed this by asking whether there was any predictability in the data (and model) using autocorrelation calculations. Our results suggest that no such predictability exists, which from a practical perspective raises questions regarding our potential to identify ‘problem’ subtypes likely to arise.

## Materials and Methods

### Data

**Figure 1:**
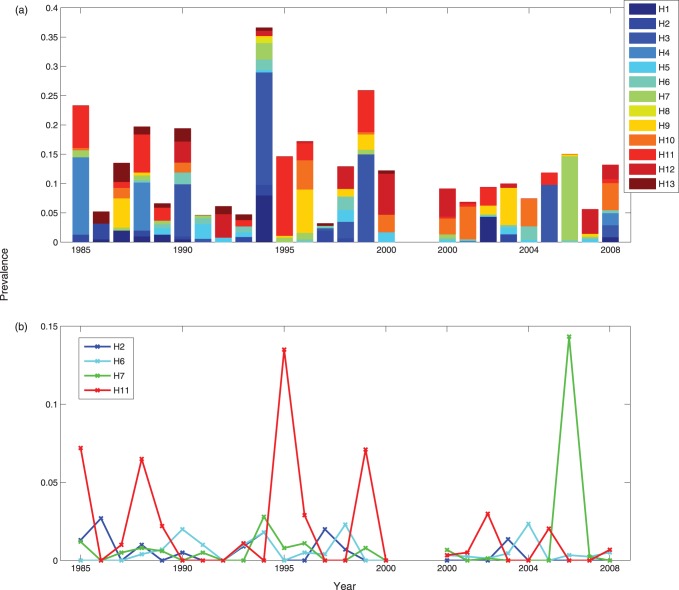
Prevalence plots of datasets 1 and 2. (a) A stacked bar chart of the approximate prevalence of HA subtypes. (b) The prevalence time series from both datasets for the four selected hemagglutinin (HA) subtypes. In both cases, the two datasets are separated by a gap on the x-axis. For dataset 1, a total of 4266 fecal or cloacal samples were collected over the time period 1985–2000 [9]; for dataset 2 the average annual sample size was 400.

We examined data collected from ruddy turnstones in Delaware Bay during May/June over multiple consecutive years. The data are from two independent sources and so we compared, not combined, them. The longer time series (1985–2000) consists of environmental fecal swabs and is estimated from data published in [9] (hereafter we refer to these data as “Dataset 1”); the more recent data (2000–2008) are partially published in [19] (these are henceforth referred to as “Dataset 2”) and consist of cloacal swabs taken from captured birds. We focused our attention on the four most frequently occurring subtypes from Dataset 1 (i.e. that are non-zero for the most number of years). The data from both sources are shown in figure 1; panel (a) is a stacked bar graph showing the (approximate) prevalence of all subtypes and panel (b) shows the (approximate) prevalence time series for the four subtypes of interest.

We conducted a series of analyses on both datasets to establish whether any patterns are apparent pertaining to either subtype dominance or diversity, and hence whether we could identify mechanisms (using a mathematical model) capable of reproducing those patterns. We used several metrics to measure subtype dominance and diversity – Simpson’s diversity index [20], change in rank vs rank (*Barton et al., unpub.*; see 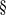 Analysis of empirical patterns) and rank-abundance [21]. We observed three distinct patterns in the data and, through the use of a mechanistic transmission model, demonstrated that two of them are consistent with a neutral model.

### Mechanistic Model

To investigate the mechanisms consistent with the diversity and dominance measures quantified in our data, we developed a stochastic transmission model, solved using Gillespie’s 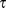-leap algorithm [22], [23]. The model incorporated those key features previously identified as important in Delaware Bay [17] and was comprised of four subtypes, multiple geographical sites and three host species: i) a duck species resident in Delaware Bay (assumed to be American black ducks - *Anas rubripes*), ii) a migratory duck species that breeds in Canada and winters in Delaware Bay (assumed to be mallards - *Anas platyrhynchos*) and iii) ruddy turnstones, a shorebird species that winters in Brazil and breeds in the Canadian Arctic, briefly resting in Delaware Bay during their spring migration. A full description of the model is provided in File S1. Note that our model permitted co-infection, but for tractability, any host may be simultaneously infected with a maximum of two subtypes [24]. Host species are assumed equally competent for all subtypes. We assumed subtype-specific immunity to wane, the rate of which was quantified in previous work [17]. We examined the sensitivity of our conclusions to this (and other) parameter choices.

We considered two possible routes of AIV transmission - direct and environmental [25], [26]. The direct route assumed a short time scale for transmission from an infected to a susceptible host and required both to concurrently inhabit the same spatial location. The environmental transmission route, however, did not assume co-location as transmission occurs indirectly, via an environmental reservoir. It has long been known that AIVs can persist for extended periods of time in water [27], [28], thus virus deposition in the environment by an infected bird may lead to consumption by a susceptible and subsequent infection without either bird interacting directly. The model, therefore, allowed for the subtype patterns to be driven by either viral persistence in the environment or through direct transmission between individuals, or a combination of both. We modelled the rate of decay of the virus in the environment as a seasonal parameter based on local temperature. We assumed the decay rate takes either a summer or winter value in each location (as in [26]) - the details are presented in File S1 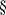 S.2.2. In modelling the environmental transmission rate, we followed [14] and ensured the neutrality of viral subtypes in our system [29]. Cross-immunity was assumed to act on the transmission probability.

Consistent with avian life history, the model was comprised of multiple components that veary seasonally. As mentioned above, seasonal migration is modelled in two of the host species with only American black ducks assumed not to migrate. All three host species exhibit seasonal hatching [30]–[32], and the duck species show patterns of seasonal mortality due to hunting [33], [34]. The final form of seasonality included in the model acts on the direct transmission terms, which we assumed to vary through the year - either due to increased territoriality (leading to reduced contact rates), as in the duck species [30], [32] - or, for ruddy turnstones, due to increased density (increasing contact rates) while in Delaware Bay [16]. Further details concerning these seasonal drivers are provided in File S1 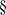 S.2.3–S.2.5.

For ruddy turnstones, we calculated the time series of prevalence for each subtype and, in order to mimic the process of field isolation, we then randomly sampled from simulated data. We assumed sampling over a 2 week time period, with the prevalence being the sum of all infected birds over that time frame divided by the total number of birds tested. We then sampled from our ‘true’ prevalence using a binomial distribution and assuming 400 birds sampled a year (to match the average annual sample size from Dataset 2).

**Figure 2:**
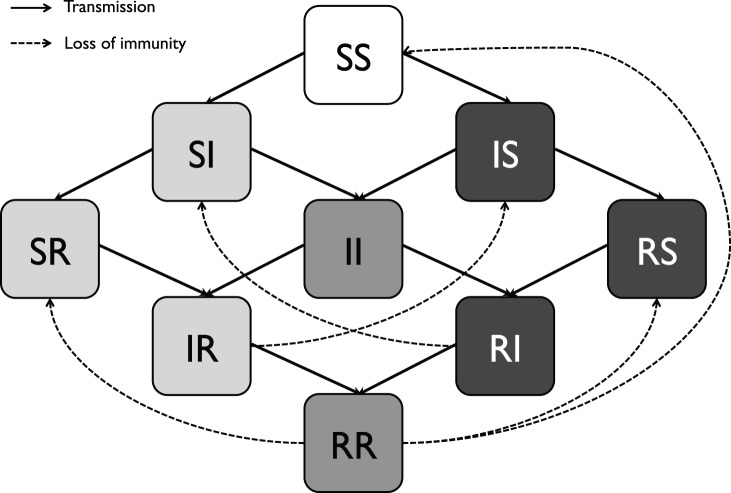
Schematic of the model with two-subtypes depicted for illustration. Hosts are born susceptible to both subtypes (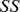) and their subsequent status with respect to both subtypes is tracked. Infection events are represented by solid arrows while loss of immunity is depicted by dotted arrows.

For clarity, a schematic of a two-subtype version of the model is shown in figure 2 - this scales up to four-subtypes as would be expected from this schematic, with the exception that we constrained co-infections to be with no more than 2 subtypes simultaneously. The full set of mean field equations underlying our stochastic model are given in 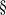 S.2.1; for clarity we present a two-pathogen system corresponding to figure 2. This scales directly to four subtypes and three hosts.

As part of our analysis, we computed Barycentric coordinates for both the model and empirical data sets. The Barycentric coordinate system is a useful tool when considering multi-subtype data. The coordinate space consists of a simplex that may be of any dimension, with each vertex corresponding (in our case) to each subtype (see figure S1). The coordinates are then calculated as the relative prevalence of each subtype, with each coordinate in Barycentric space calculated by dividing the prevalence of a given subtype by the sum of subtype prevalences (see File S1 for more details). By employing this coordinate system it is possible to consider changing dominance in subtype space through time.

## Results

### Analysis of Empirical Patterns

**Figure 3:**
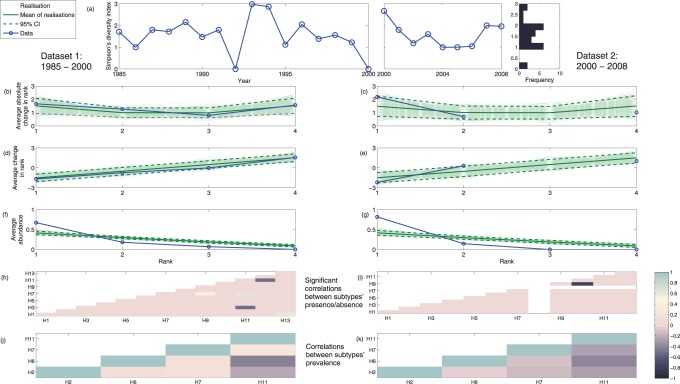
Plots showing analyses of dominance and diversity patterns from datasets 1 and 2. The Simpson’s diversity index for the four subtypes of interest from both datasets is presented in (a). Absolute change in rank against rank ((b) and (c)), change in rank against rank ((d) and (e)) and rank-abundance curves ((f) and (g)) are presented for each dataset - panel letters given refer to datasets 1 and 2 respectively. Panels (h)-(k) show correlations in the data, with (h)-(i) showing any significant correlations between subtype presence/absence for the complete datasets, and (j)-(k) showing any correlations between prevalence levels for the 4 subtypes of interest.

Our first empirical result concerned Simpson’s diversity index, which gives a time-dependent measure of both diversity and dominance. As shown in figure 3(a), in both sets of data, there was notable temporal variation in diversity through time. Its accompanying histogram shows however that although there may be considerable year-to-year variability in this metric, overall the majority of values fall into a subset of the full range. Our second empirical result concerned the rank-specific change in rank from year to year. To measure change in rank vs rank, each of the four subtypes was annually assigned a rank between 1 and 4, with 1 the most dominant and 4 the least dominant. We repeated this for each ranking and averaged over the duration of the time series to generate a change in rank vs. rank curve. These curves are then contrasted with randomly generated test data (see File S1 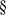 S.1 for a detailed description). As shown in figures 3(b)–(e), in both empirical data sets, the change in rank vs rank and the absolute change in rank vs rank fall within the 95% confidence intervals for the simulated random data. Our third empirical result for dominance concerns rank-abundance, which is well-known in ecology and quantifies the average relative dominance of each subtype over the time series. The rank-abundance curves for each dataset (figure 3(f),(g)) deviate from random, with the top ranked subtype, on average, more abundant in the data than would be predicted from a random sample.

As a further study, we looked for correlations both between the presence/absence of subtypes and between the prevalence levels of subtypes to identify possible inter-subtype relationships. Figures 3(h) & (i) demonstrate very few significant correlations. When we focused on the four subtypes of particular interest and plotted the correlation between prevalence levels, we found that while many were negatively correlated, none were statistically significant. This is to some extent supported by the rank-abundance curve, which suggests that subtypes of high rank have high prevalence relative to the other subtypes. This lack of correlation between subtype presence/absence also suggests that, in ruddy turnstones, there may not be a significant role for heterotypic immunity - a suggestion we further investigated using the stochastic transmission model.

This mix of apparently random and non-random patterns raises an obvious question - what (if any) is the role of cross-immunity in this system? To address this, we used the transmission model to tease apart the relative impacts of hetero-subtypic immunity from other epizootiological factors that may contribute to the observed dynamics.

### Modelling Findings

**Figure 4:**
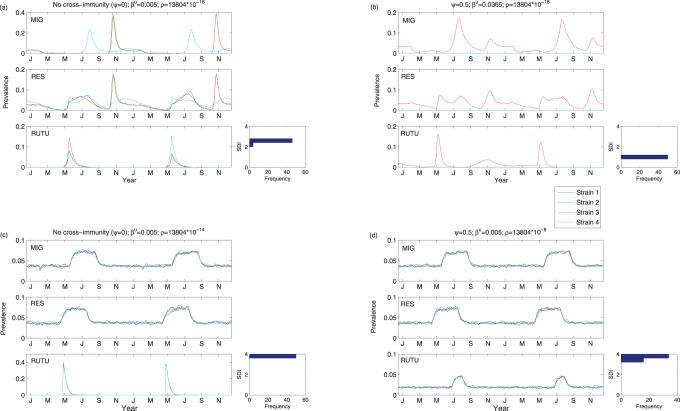
Model predicted prevalence curves for all three hosts for a variety of different parameter sets. Panel (a) shows the prevalence curves for migratory ducks, resident ducks and ruddy turnstones for no cross-immunity and low transmission and consumption rates. Panel (b) shows the prevalence curves for all species for a cross-immunity rate of 0.5, with a higher transmission rate than in (a) and with a low consumption rate. Panel (c) shows the case with no cross-immunity, low transmission rate and an increased consumption rate (over (a) and (b)). Finally, panel (d) shows the prevalence curves when the cross-immunity rate is 0.5, transmission rate is low and consumption rate is greatly increased. In each panel, the histogram next to the ruddy turnstone (RUTU) prevalence curve is the histogram of the Simpson’s diversity index (SDI), as averaged over all simulations with the given parameter set. Simpson’s diversity index is calculated from a sample of the true prevalence, as calculated while the birds are present in Delaware Bay. See model description for more details on sampling. Note that “Mig” here stands for migrating ducks and “Res” denotes resident ducks.

Using the model described in “Material and Methods - Mechanistic Model”, we examined the role of direct transmission in ruddy turnstones, consumption rate (varied uniformly across all three host species) and cross-immunity (also varied across all host species). In all cases, we maintained identical subtype parameters (i.e. neutrality) to establish the role of stochasticity in the system. Some sample prevalence curves are depicted in figure 4 and demonstrate changes in model behaviour under different parameterization. In particular, modifying the consumption rate can drive radically different behaviour, with non-zero prevalence observed year-round in the duck species (figure 4(c)). Furthermore, it is apparent from these snapshots that stochasticity alone can promote changes in rank from year to year between the subtypes.

**Figure 5:**
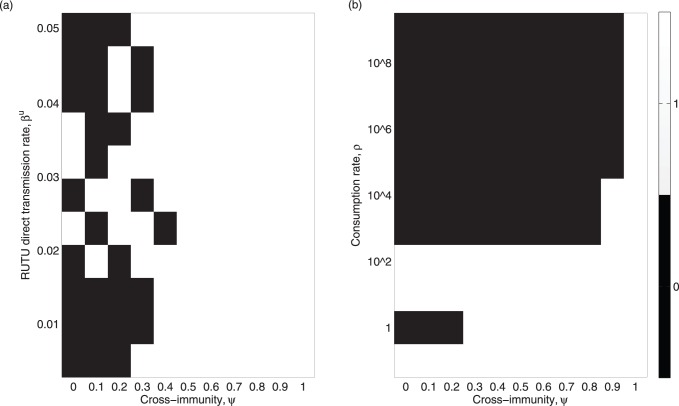
Plots showing the Kolmogorov-Smirnov test results from comparing histograms of Simpson’s diversity index from the datasets and the model. The histograms consist of 10 equal sized bins between 0 and 4 for both the datasets and the model. Panels (a) and (b) show whether or not the null hypothesis (that the histograms come from the same distribution) can be rejected -the result is one if it can be rejected, 0 otherwise. Panel (a) shows the results for varying cross-immunity and direct transmission rate in ruddy turnstones; (b) shows the results for varying cross-immunity and consumption rate. For each parameter set, the model histogram was constructed from the mean Simpson’s diversity index, as calculated from 10 model simulations.

Next, we measured how well our model captured the changing subtype diversity observed in the data. We compared a histogram of Simpson’s diversity index from the data with one generated by our model, using a two-sample Kolmogorov-Smirnov test [35] to assess whether the two histograms belonged to the same distribution. In particular, we systematically varied the strength of cross immunity (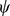), the direct transmission rate 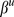 and enviromental consumption rate 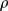 with the aim of exploring parameter regions that generated dynamics consistent with data. As shown in figure 5, the null hypothesis that histograms from our model and data arose from the same distribution could be rejected either when subtype cross-immunity was very strong or when environmental transmission was very low. On this first measure, at least, we identified that parameter combinations exist under which a neutral model with demographic stochasticity and the absence of immune-mediated dynamics could produce results with the same statistical signatures as the data.

**Figure 6:**
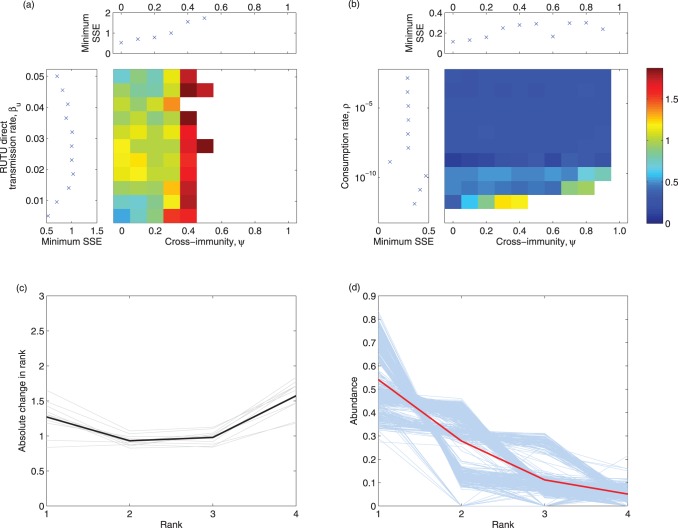
Synthetic likelihoods comprising of change in rank vs. rank and rank-abundance curves between the model and data. The heat maps in panels (a) and (b) depict 2-D likelihood profiles, with single pararmeter profiles shown in upper and left panels. The plots show the normalised SSEs for varying cross-immunity and either the ruddy turnstone transmission rate (a) or the consumption rate (b) against the metrics absolute change in rank vs. rank and rank-abundance. White space in both plots is the result of subtype extinctions leading to much reduced fits to the data (for more information on average number of subtypes coexisting for different parameter sets, see File S1). Panels (c) and (d) give the absolute change in rank vs rank (c) and rank-abundance curves (d) for the best fit estimate (as judged from the SSEs). Lighter lines (grey in (c), blue in (d)) show the results from individual realisations and darker lines (black in (c), red in (d)) show averages.

To evaluate further model parsimony with data, we estimated a synthetic likelihood [36], which quantifies model fit using statistical descriptors, or probes. Specifically, focusing on the change in rank vs rank and rank-abundance associations, we calculated sum of squared errors (SSEs) for model ouput against dataset 1 for both metrics, with values normalised and summed to provide a combined measure of model fit. For each set of parameter values, we averaged the results over 10 model simulations. The heat maps presented in Figures 6(a) and (b) depict 2-D synthetic likelihood profiles, with panels above and to the left summarizing profiles over cross-immunity and transmision rate (in Fig. 6a) and consumption rate (in Fig. 6b), respectively. Overall, these calculations indicate that best-fit parameters assume no cross-immunity, low direct transmission and a moderate consumption rate. Figures 6(c) and 6(d) present the results of both measured metrics (absolute change in rank against rank and rank-abundance) for the best-fit parameters. Crucially, these results indicate that stochasticity alone is capable of generating both the dominance and diversity patterns observed in these data. For comparison, we include figures equivalent to figure 6 for each metric individually in File S1 (figures S3– S5).

**Figure 7:**
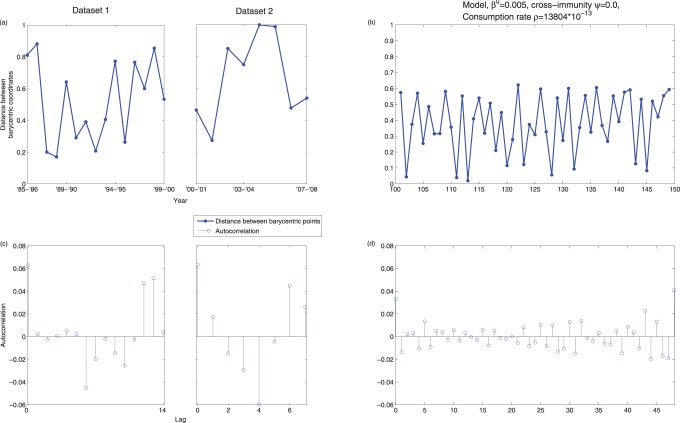
The Cartesian distance between Barycentric coordinates for both the model and data, and their respective autocorrelations. Figures showing the Cartesian distance between Barycentric coordinates for both the data (a) and a model simulation using the best-fit parameter set (c). The autocorrelations for both of these are shown in figures (b) and (d) respectively. The 95% significance levels are not shown as their threshold is much larger than the calculated values.

Finally, we examined the correlation structure in subtype fluctuations as quantified using Barycentric ordination. In particular, for each data set we calculated the distance between successive Barycentric coordinates, which provides information regarding the shifting subtype dominance through time (see ‘Materials and Methods - Mechanistic Model’ and File S1 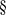 S.4, figures S6 & S7 for additional details). Small estimated distances would indicate a temporally stable relative subtype composition, while large values would suggest dramatic changes in relative prevalences from year to year. In Figures 7(a) and 7(c) we present Barycentric distances for both datasets together with estimated autocorrelation functions (ACFs). Figures 7(b) and (d) depict parallel plots for model output using best-fit parameters. For both model and data, the peak autocorrelation functions occur at lag 0 and rapidly fall well below the 95% significance level. These ACF patterns indicate little predictability in changing subtype dominance through time - consistent with patterns that are random.

## Discussion

Our work here offers new insights into the epizootiology of AIVs in wild bird populations. We have addressed some of the open questions surrounding the maintenance of multiple subtypes in wild birds, focusing on ruddy turnstones in Delaware Bay as a species that has the potential to be a key player in AIV dynamics in North America [17]. Concentrating on diversity and dominance measures meant that we could address particular questions relating to the relative importance of the environmental reservoir versus the direct transmission route, the role of cross-immunity in the system and how subtype differences contributed to the observable dynamics.

Data analyses from two independent datasets indicate that randomness plays a role in the observed subtype dynamics. In both data sets, the change in rank from one year to the next is not different from randomly generated data with little or no correlation structure. When considering the rank-abundance curves, however, the data are clearly at odds with the random prediction. Together, these results suggest that, while there may be little inter-annual information concerning subtype ranking, it may be possible to infer information on the relative prevalence of subtypes at different ranks.

Applying these ranking metrics to the transmission model highlights the role played by demographic stochasticity. Assuming identical subtype parameters, we have shown that stochasticity alone can lead to variance in rank from year to year that is consistent with data. More surprising, our results indicate that our stochastic model is capable of generating the empirical non-random rank-abundance curve. Furthermore, a neutral model is sufficient to create a plausible diversity pattern in the model results, as quantified by Simpson’s diversity index. Together, these results imply that it may not be necessary to invoke differences between subtypes in order to explain empirical patterns. This theoretical result supports recent experimental work suggesting that subtype differences (in ducks) do not play a significant role in either viral shedding or persistence in the environment [37]. Moreover, the compatibility between our theoretical and empirical results demonstrates that it is possible to use information at multiple scales to make inferences about the system as a whole. Here, we have taken population-level data and, using a population-level model, have proposed putative mechanisms that may contribute to the coexistence of multiple subtypes within a wild bird population. We find that these mechanisms (in particular, that subtype differences are not necessary to reproduce the statistical signatures observed in the data) are supportive of similar, empirical, results found in individual level challenge experiments [37].

Using a probe-matching approach, we found that heterosubtypic immunity is not necessary for explaining the patterns we observe in AIV dominance dynamics in ruddy turnstones. Indeed, our model predicts that strong cross-immunity will inevitably lead to subtype extinctions (figure S2). It is worth noting that homotypic immunity is included in the model - for ruddy turnstones, the duration of this immunity is, on average, 1 year [19]. Homotypic immunity may well be influencing the observed subtype dynamics, but further challenge experiments need to be conducted in ruddy turnstones to fully understand the nature and scope of that immunity. Similarly, while we find no evidence for cross-immunity in our model results, immunity lasting less than a year may well exist. The resolution of the data are too coarse to identify if this is the case - again, challenge experiments are needed to quantify the strength and duration of any short-term cross-immunity.

We find that consumption rate from the environmental reservoir can substantially impact the dynamics, with direct transmission playing a lesser role than cross-immunity. That the environmental reservoir should have such an impact is logical when we consider the relative time scales - acting over long periods of time, the environmental reservoir can maintain transmission during periods of lower numbers of susceptibles or lower contact rates. This is multifaceted, as the environmental reservoir serves as a transmission route for multiple species. Thus, the subtype composition of the environmental reservoir will be influenced, not just by ruddy turnstones, but also by other bird species (in the case of our model, this is the two duck species). Biologically, the diversity of species likely to be seeding the environmental reservoir with AIV subtypes may influence the random nature of some of the patterns observed in ruddy turnstone AIV subtype dynamics.

Overall, this work illustrates that a neutral model incorporating demographic stochasticity is capable of capturing the diversity and dominance patterns observed in the field. Equally, without sufficient uptake of virus from an environmental reservoir, the model predicted changes in rank are not consistent with the data. Finally, our attempts to estimate key parameters using statistical descriptors of the data did not identify a role for cross-immunity. The failure to detect any year-to-year autocorrelation in Barycentric coordinates provided additional evidence that there is little or no inertia in subtype composition (Fig. 5). This conclusion is reenforced in our model, assuming best-fit parameters. Crucially, from a practical perspective, unpredictability in subtype dominance dynamics may clearly impede efforts to anticipate which subtype has the potential to be the greatest risk (due to high prevalence levels) from year to year.

## Supporting Information

Figure S1 **Barycentric coordinate system in 2-D.** The Barycentre is marked with a red star.
(EPS)

Figure S2 **Average number of subtypes present (over 10 realisations) for each set of parameter values.**
(EPS)

Figure S3 **Sample prevalence curves from the model for the best fit parameter set.** The panels (from top to bottom) show prevalence curves for all four subtypes in migrating ducks, resident ducks and ruddy turnstones (RUTUs).
(EPS)

Figure S4Panels (a) and (b) show the normalised SSEs for varying cross-immunity and either the ruddy turnstone transmission rate (a) or the consumption rate (b) against the metric absolute change in rank vs. rank. Panels (c) and (d) give the absolute change in rank vs rank curves for the best fit SSE parameters for either (c) ruddy turnstone direct transmission rate 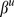 or (d) consumption rate 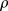. These occur at either a medium level of cross-immunity (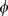 = 0.5) and consumption rate (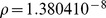), or with low values for cross-immunity (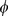 = 0) and direct transmission rate (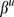 = 0.005).(EPS)

Figure S5Panels (a) and (b) show the normalised SSEs for varying cross-immunity and either the ruddy turnstone transmission rate (a) or the consumption rate (b) against the metric rank-abundance. Panels (c) and (d) give the rank-abundance curves for the best fit SSE parameters for either (c) ruddy turnstone direct transmission rate 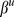 or (d) consumption rate 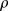. These occur at a low level of cross-immunity (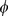 = 0.2), with a low value for consumption rate (panels (b),(d); 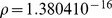) or a high value for direct transmission rate (panels (a), (c); 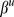 = 0.0365).(EPS)

Figure S6 **Lagged scatterplots showing for datasets 1 (a) and 2 (b), with linear fits also shown.** Correlation coefficients are given above each plot and show none of the correlations are significant.
(EPS)

Figure S7 **Change in rank ((a) and (b)) and rank abundance ((c) and (d)) plots for both high transmission potential ((a) and (c)) and low transmission potential ((b) and (d)).**
(EPS)

File S1 (PDF)
