# Supplementary material for: Neutrality, Cross-Immunity and Subtype Dominance in Avian Influenza Viruses
Source: PLoS One. 2014 Feb 25;9(2):e88817. doi: 10.1371/journal.pone.0088817 (PMC3934864; doi:10.1371/journal.pone.0088817)
Supplement: File S1 — (PDF) [file pone.0088817.s008.pdf]

S Supplementary Information

S.1 Data Analysis

To determine the randomness of the datasets in relation to various metrics, we constructed a sample of 1000 random time series for each dataset, with the random time series the same length as the dataset in question. Values for the random time series were drawn from a uniform distribution and could take values on the interval [0, 0.25]. The upper limit was chosen to maintain similarity in scale between the observed prevalence levels in Datasets 1 and 2.

S.2 Model Details

As described in the main text, this model is a multi-site, multi-species, multi-subtype model that details the interactions of three species that use Delaware Bay for some or all of the year. The basic structure for a single subtype version of this model is given in [1]; here we extend that to four subtypes. The assumptions surrounding the model are presented in the main text.

S.2.1 Model Equations

$$\frac{\mathrm{d}SS}{\mathrm{d}t} = b(t)N - (\beta_1 I_1 + \delta\rho_1\left(\frac{\rho_1 V_1}{\rho_1 V_1 + \kappa_1}\right)V_1 + \beta_2 I_2 + \delta\rho_2\left(\frac{\rho_2 V_2}{\rho_2 V_2 + \kappa_2}\right)V_2)SS + \epsilon_1 RS + \epsilon_2 SR, \tag{S1a}$$

$$\frac{\mathrm{d}IS}{\mathrm{d}t} = (\beta_2 I_2 + \delta\rho_2\left(\frac{\rho_2 V_2}{\rho_2 V_2 + \kappa_2}\right)V_2)SS - \gamma_1 IS - (1-\psi_2)(\beta_2 I_2 + \delta\rho_2\left(\frac{\rho_2 V_2}{\rho_2 V_2 + \kappa_2}\right)V_2)IS + \epsilon_2 IR, \tag{S1b}$$

$$\frac{\mathrm{d}SI}{\mathrm{d}t} = (\beta_1 I_1 + \delta\rho_1\left(\frac{\rho_1 V_1}{\rho_1 V_1 + \kappa_1}\right)V_1)SS - \gamma_2 SI - (1-\psi_1)(\beta_1 I_1 + \delta\rho_1\left(\frac{\rho_1 V_1}{\rho_1 V_1 + \kappa_1}\right)V_1)SI + \epsilon_1 RI, \tag{S1c}$$

$$\frac{\mathrm{d}II}{\mathrm{d}t} = (1-\psi_1)(\beta_1 I_1 + \delta\rho_1\left(\frac{\rho_1 V_1}{\rho_1 V_1 + \kappa_1}\right)V_1)SI + (1-\psi_2)(\beta_2 I_2 + \delta\rho_2\left(\frac{\rho_2 V_2}{\rho_2 V_2 + \kappa_2}\right)V_2)IS - (\gamma_1 + \gamma_2)II, \tag{S1d}$$

$$\frac{\mathrm{d}RI}{\mathrm{d}t} = \gamma_1 II + (1-\psi_2)(\beta_2 I_2 + \delta\rho_2\left(\frac{\rho_2 V_2}{\rho_2 V_2 + \kappa_2}\right)V_2)RS - \gamma_2 RI - \epsilon_1 RI, \tag{S1e}$$

$$\frac{\mathrm{d}IR}{\mathrm{d}t} = \gamma_2 II + (1-\psi_1)(\beta_1 I_1 + \delta\rho_1\left(\frac{\rho_1 V_1}{\rho_1 V_1 + \kappa_1}\right)V_1)SR - \gamma_1 IR - \epsilon_2 IR, \tag{S1f}$$

$$\frac{\mathrm{d}RS}{\mathrm{d}t} = \gamma_1 IS - (1-\psi_2)(\beta_2 I_2 + \delta\rho_2\left(\frac{\rho_2 V_2}{\rho_2 V_2 + \kappa_2}\right)V_2)RS + \epsilon_2 RR - \epsilon_1 RS, \tag{S1g}$$

$$\frac{\mathrm{d}SR}{\mathrm{d}t} = \gamma_2 SI - (1-\psi_1)(\beta_1 I_1 + \delta\rho_1\left(\frac{\rho_1 V_1}{\rho_1 V_1 + \kappa_1}\right)V_1)SR + \epsilon_1 RR - \epsilon_2 SR, \tag{S1h}$$

$$\frac{\mathrm{d}RR}{\mathrm{d}t} = \gamma_1 IR + \gamma_2 RI - (\epsilon_1 + \epsilon_2)RR, \tag{S1i}$$

$$\frac{\mathrm{d}V_1}{\mathrm{d}t} = \omega_1 I_1 - \eta_1 V_1, \tag{S1j}$$

$$\frac{\mathrm{d}V_2}{\mathrm{d}t} = \omega_2 I_2 - \eta_2 V_2. \tag{S1k}$$

Here $I_1 = IS + II + IR$, $I_2 = SI + II + RI$ and $\delta_i = \frac{(V_i/\kappa_i)}{(V_1/\kappa_1)+(V_2/\kappa_2)}$, $i = 1, 2$. The parameters are described in the following sections. The class names follow the standard SIR format, with S representing susceptibles, I representing infecteds and R representing immune classes. The first letter of each variable represents the infection status for subtype 1, and the second letter represents the infection status for subtype 2 (so, for example, class SI tells us that individuals

in this class are susceptible to subtype 1 and infected with subtype 2). Classes V_i, $i = 1, 2$ represent the environmental reservoir for each subtype. For a more thorough description of the single subtype system, see [1].

The parameters described in §S.2.2-§S.2.5 apply to all subtypes, as we assume all subtype parameters are identical in this study. These sections, and table S-1, have previously been published in [1]; the table appeared in the main text and the sections appeared in the Electronic Supporting Information.

S.2.2 Transmission parameters

Transmission within species is denoted by β^{ii}, $i = u, r, m$ and between species as β^{ij} for $i \neq j$, $i, j = u, r, m$. We assume that the transmission between species (β^{mr}, β^{rm}, β^{ur} and β^{ru}) is the mean of the average direct transmission rates (e.g. $\beta^{mr} = \beta^{rm} = \frac{1}{2}(\beta_0^m + \beta_0^r)$) within both species. Between-species transmission rates are non-zero only when the migrating species are present in Delaware Bay and interacting with the resident ducks. These periods of time are given by the migration parameters in Section S.2.3. The resulting transmission matrix is

$$\beta = \begin{pmatrix} \beta^{uu} & 0 & \beta^{ur} \\ 0 & \beta^{mm} & \beta^{mr} \\ \beta^{ru} & \beta^{rm} & \beta^{rr} \end{pmatrix} = \begin{pmatrix} \beta^{uu} & 0 & \frac{1}{2}(\beta_0^u + \beta_0^r) \\ 0 & \beta^{mm} & \frac{1}{2}(\beta_0^m + \beta_0^r) \\ \frac{1}{2}(\beta_0^u + \beta_0^r) & \frac{1}{2}(\beta_0^m + \beta_0^r) & \beta^{rr} \end{pmatrix}. \tag{S2}$$

We assume perfect mixing between resident ducks and either of the species they interact with; for migratory ducks this is clear from the similarity of their habitats and behaviours. Between resident ducks and ruddy turnstones, we base this on research that has shown a variety of habitats are important to shorebird species [2], offering them the opportunity to interact with other species.

The transmission parameters for within-species transmission are given by

$$\beta^{ii} = \beta_0^i \frac{(1 + \beta_1^i Breeding_i(t))}{\frac{1}{365}((1 + \beta_1^i)D^+ + (1 - \beta_1^i)D^-)}, \tag{S3}$$

with D^+ denoting the number of days of high transmission and D^- denoting the number of days of low transmission, $i = u, r, m$. The denominator of (S3) ensures that the average of β^{ii}, $\bar{\beta^{ii}}$, is equal to β_0^i. The 'Breeding season' parameters for the migrating and resident ducks are presented below.

$$Breeding_m(t) = \begin{cases} -1 & \text{if } 0.25 < t < 0.42, \\ 1 & \text{otherwise.} \end{cases} \tag{S4}$$

$$Breeding_r(t) = \begin{cases} -1 & \text{if } 0.21 < t < 0.38, \\ 1 & \text{otherwise.} \end{cases} \tag{S5}$$

We use the '$Mixing_u$' function (given in Section S.2.3) in place of a '$Breeding_u$' function in the ruddy turnstone's transmission term - this is the period of time ruddy turnstones are present in Delaware Bay and is given in (S7).

S.2.3 Migration parameters

The parameters determining the location of each species at each point in time (i.e. relating to migration patterns) are given below; the '$Mixing$' and '$Mixing_u$' parameters give the times the migrating ducks and ruddy turnstones respectively are in Delaware Bay. The '$Mixing_u$' parameter is also used to calculate the within-species direct transmission rate for ruddy turnstones (as described in Section S.2.2). The '$Summer$' and '$Wintering$' parameters give the periods when the ruddy turnstones are on their summer and winter grounds.

$$Mixing(t) = \begin{cases} 0 & \text{if } 0.125 < t < 0.8, \\ 1 & \text{otherwise.} \end{cases} \tag{S6}$$

$$Mixing_u(t) = \begin{cases} 1 & \text{if } 0.354 < t < 0.417, \\ 0 & \text{otherwise.} \end{cases} \tag{S7}$$

$$Wintering(t) = \begin{cases} 0 & \text{if } 0.354 < t < 0.683, \\ 1 & \text{otherwise.} \end{cases} \tag{S8}$$

$$Summer(t) = \begin{cases} 1 & \text{if } 0.417 < t < 0.683, \\ 0 & \text{otherwise.} \end{cases} \tag{S9}$$

The shedding and consumption rates for ruddy turnstones and migrating ducks for each location they visit are also defined using the above parameters (as the above parameters determine which location each species in for every point in time), so for example shedding by the migrating ducks in Delaware Bay, ω^{mdb}, uses the $Mixing(t)$ parameter - $\omega^{mdb} = \omega^m Mixing(t)$ (see Table 1 for ω^m; other shedding and consumption rate parameters are constructed similarly).

S.2.4 Hatching and mortality parameters

The hatching parameter for each species is given below. The superscripts u, r, m denote ruddy turnstones, resident ducks and migrating ducks respectively. These parameters (in the case of hatching) define when hatching occurs in the model - i.e. the periods of time when it is non-zero. In the case of the mortality parameters, the '$Death^i(t)$' $(i = m, r)$ give the step functions used for the mortality rates in both duck species (so $\mu^m = Death^m(t)$; $\mu^r = Death^r(t)$).

$$b^m(t) = \begin{cases} b_0^m & \text{if } 0.42 < t < 0.67, \\ 0 & \text{otherwise.} \end{cases} \tag{S10}$$

$$b^r(t) = \begin{cases} b_0^r & \text{if } 0.33 < t < 0.58, \\ 0 & \text{otherwise.} \end{cases} \tag{S11}$$

$$b^u(t) = \begin{cases} b_0^u & \text{if } 0.522 < t < 0.622, \\ 0 & \text{otherwise.} \end{cases} \tag{S12}$$

The seasonal mortality rates for the duck species (as a result of hunting [3, 4, 5]) are given below. Subscripts r, m denote resident and migratory ducks respectively.

$$Death^m(t) = \begin{cases} 0.25 & \text{if } 0.02 < t < 0.83, \\ 1.57 & \text{otherwise.} \end{cases} \tag{S13}$$

$$Death^r(t) = \begin{cases} 0.3 & \text{if } 0.02 < t < 0.83, \\ 1.35 & \text{otherwise.} \end{cases} \tag{S14}$$

S.2.5 Virus durability parameters

The persistence terms for virus in the environment (these are defined as η^k, $k = b, db, a$) are time-dependent (as they rely on temperature [6]), taking a 'winter' value and a 'summer' value in Delaware Bay and the breeding grounds for both migrating ducks and ruddy turnstones. They are calculated from weather data in the different locations [7, 8, 9] and using [6] to convert this to virus persistence in the environment. The virus persistence at the ruddy turnstones' wintering ground (assuming to be coastal Brazil [10]) is taken to constant ($\eta^w = 167.9$, superscript w represents

wintering ground) throughout the year as the region has very little variation in temperature [11]. The functions for persistence rate of virus at the duck breeding grounds, Delaware Bay and the ruddy turnstone breeding grounds (denoted by superscripts b, db and t respectively) are

$$\eta^b(t) = \begin{cases} 24.3 & \text{if } 0.33 < t < 0.83, \\ 4.9 & \text{otherwise,} \end{cases} \tag{S15}$$

$$\eta^{db}(t) = \begin{cases} 42.6 & \text{if } 0.33 < t < 0.83, \\ 13.9 & \text{otherwise} \end{cases} \tag{S16}$$

and

$$\eta^t(t) = \begin{cases} 10 & \text{if } 0.33 < t < 0.83, \\ 1.6 & \text{otherwise} \end{cases} \tag{S17}$$

respectively.

S.3 Barycentric coordinate system

The Barycentric coordinate system was invented by Möbius in 1827 [20]. It provides an alternative coordinate system, based on a simplex (of any dimension), that allows us to consider data in a different way. In this coordinate system each vertex represents a particular quantity. Residing at any of the vertices shows absolute domination by that quantity, with the other quantities equal to zero. Through normalisation, each vertex has coordinate value one for that quantity and zero for the other quantities represented. The Barycentre is the centre of the simplex and represents equality between all quantities, thereby having coordinate value of $1/3$ for each quantity. The easiest simplex to consider (for explanation purposes) is the 2-D simplex, or triangle, which is a representation of three quantities. For quantities q_1, q_2, q_3, the Barycentric coordinates are thus $(\frac{q_1}{q_1+q_2+q_3}, \frac{q_2}{q_1+q_2+q_3}, \frac{q_3}{q_1+q_2+q_3})$. Figure S1 shows the idea of Barycentric coordinates, in 2-D, with the Barycentre marked.

S.4 Further explanatory figures

We provide a number of explanatory figures to expand on the model results presented in the main text. Initially we consider the average number of strains present (over 10 simulations) for each parameter set tested (c.f. figure 4(a) and (b)). This results offers greater insight into why

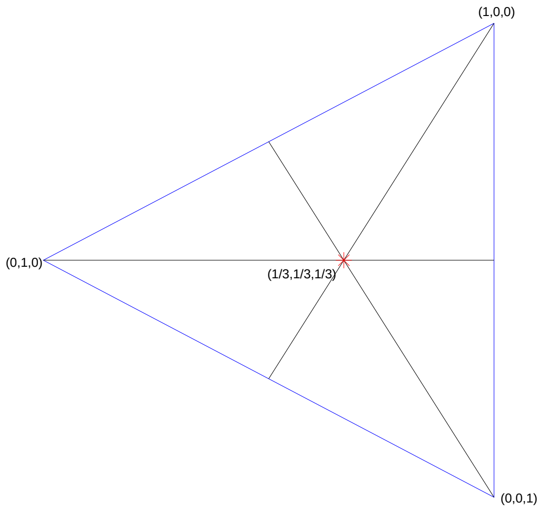

Figure S1. Barycentric coordinate system in 2-D. The Barycentre is marked with a red star.

not all parameter sets provide estimates in figure 4 - it is that the number of strains present (on average) is simply too small to allow the metric in question to be computed.

While we present the results of the best fit parameter set for the metrics change in rank and rank abundance in the main text (figure 4(c) and (d)), in figure S5 we show some sample prevalence curves, taken from one simulation using the best fit set of parameters. In particular, the bottom panel shows the prevalence curves for ruddy turnstones, and illustrates the different trajectories of each subtype modelled.

Figure 4 in the main text shows the combined results for calculating the sum of squared errors for both change in rank and rank abundance. Figures S6 and S7 show these results separately, with S6 showing the results from the change in rank metric, and S7 showing the results from the rank abundance metric. We find that the best fit parameter set for change in rank when varying the ruddy turnstone direct transmission rate and cross-immunity gives a very dissimilar result to that in the data, but that the results are much better when using the best fit parameter set from varying consumption rate and cross-immunity. Where the rank-abundance curves are concerned, both scenarios are capable of creating a curve similar to that seen in the data. When considering these individual fits, we find that in three cases, some cross-immunity is indicated (in two of the three cases, this is for a value of $\psi = 0.2$). However, when fitting both metrics together (as we do in the main text), we find that the set of parameters that give the best fit to both metrics simultaneously include no cross-immunity.

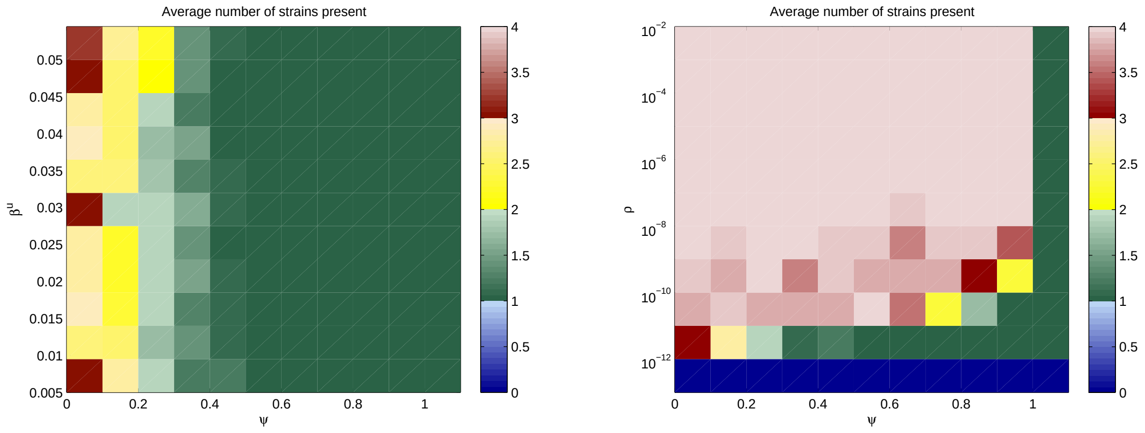

(a) (b)

Figure S2. Average number of subtypes present (over 10 realisations) for each set of parameter values.

As an extension to the autocorrelation plot (figure 5), in figure S8 we present the lagged scatterplots for both datasets. We calculate the correlation coefficient for each scatterplot and find that none are significant (thereby supporting the result in figure 5, where none of the lags cross the 95% CI).

S.5 Sensitivity analysis

To conduct a sensitivity analysis on the model, we created two scenarios - one of either 'high transmission potential' or 'low transmission potential', using the best fit parameter set predicted in the main text. In the high transmission potential scenario we increased direct transmission and consumption rates (in all species) and viral persistence in the environment by 10% and decreased the duration of immunity and recovery rates (in all species) by 10%. In the low transmission potential scenario, the increased rates were instead decreased by 10%, and the decreased rates were instead increased. The results are shown in figure S9, and we find that, in both transmission potentials, the change in rank curves follow the same basic shape. In the case of low transmission potential, the change in rank takes a somewhat higher value for ranks 1 and 4 than observed in the best fit model results, albeit close to the value given by dataset 2 for rank 1. The mean rank abundance curves are both shallower than that from the best fit model results, although (in the case of low transmission potential) there is a lot of variation between individual curves.

Together, this sensitivity analysis shows that the model has some sensitivity to the parameter set used, but is still able to recreate the change in rank patterns shown in the main text. The rank abundance curve is more variable, but (in the case of low transmission potential) maintains

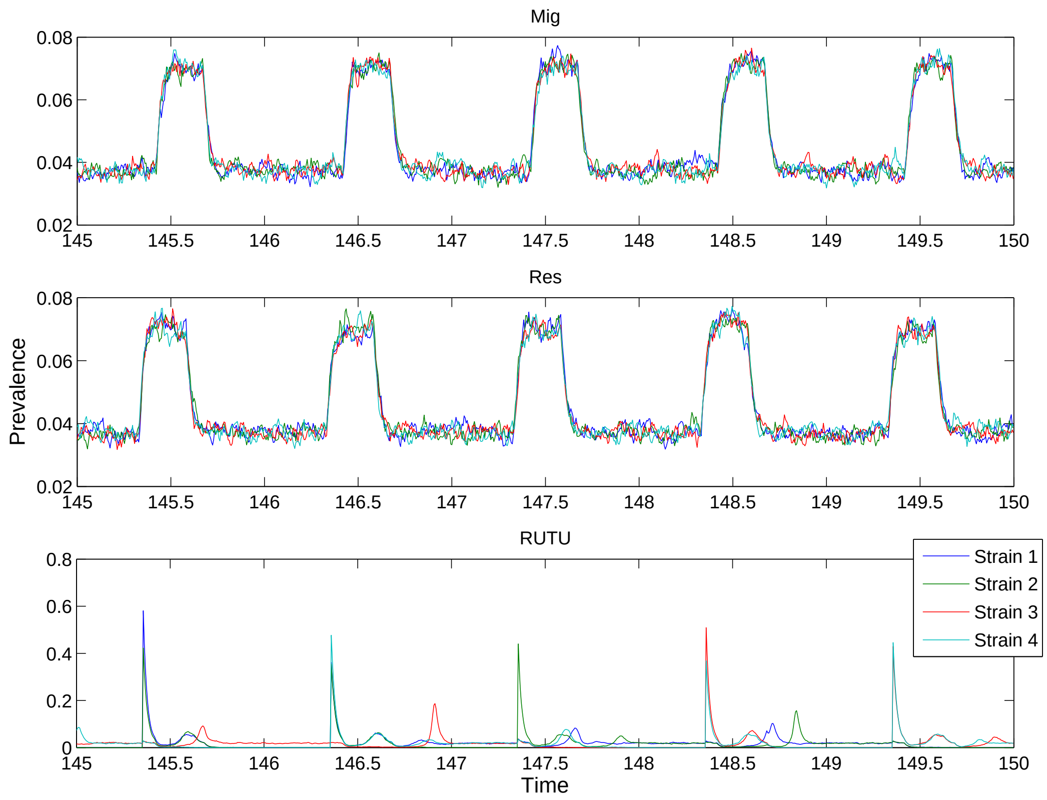

Figure S3. Sample prevalence curves from the model for the best fit parameter set. The panels (from top to bottom) show prevalence curves for all four subtypes in migrating ducks, resident ducks and ruddy turnstones (RUTUs).

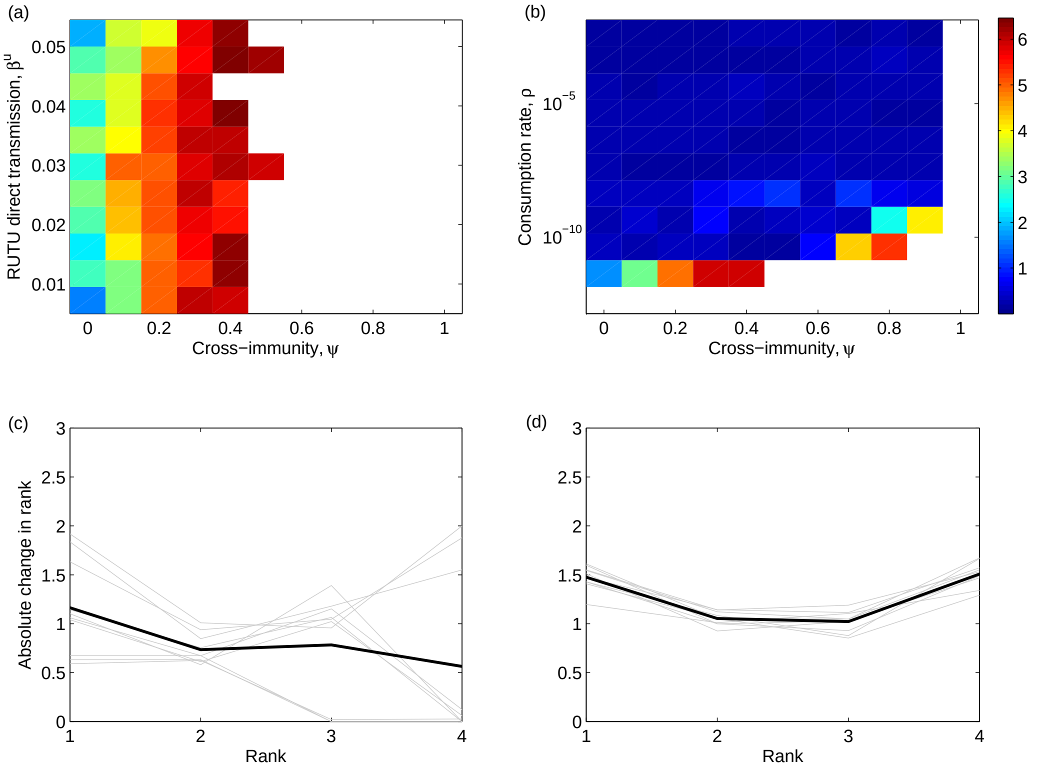

Figure S4. Panels (a) and (b) show the normalised SSEs for varying cross-immunity and either the ruddy turnstone transmission rate (a) or the consumption rate (b) against the metric absolute change in rank vs. rank. Panels (c) and (d) give the absolute change in rank vs rank curves for the best fit SSE parameters for either (c) ruddy turnstone direct transmission rate β^u or (d) consumption rate ρ. These occur at either a medium level of cross-immunity ($\psi = 0.5$) and consumption rate ($\rho = 13804 * 10^{-8}$), or with low values for cross-immunity ($\psi = 0$) and direct transmission rate ($\beta^u = 0.005$).

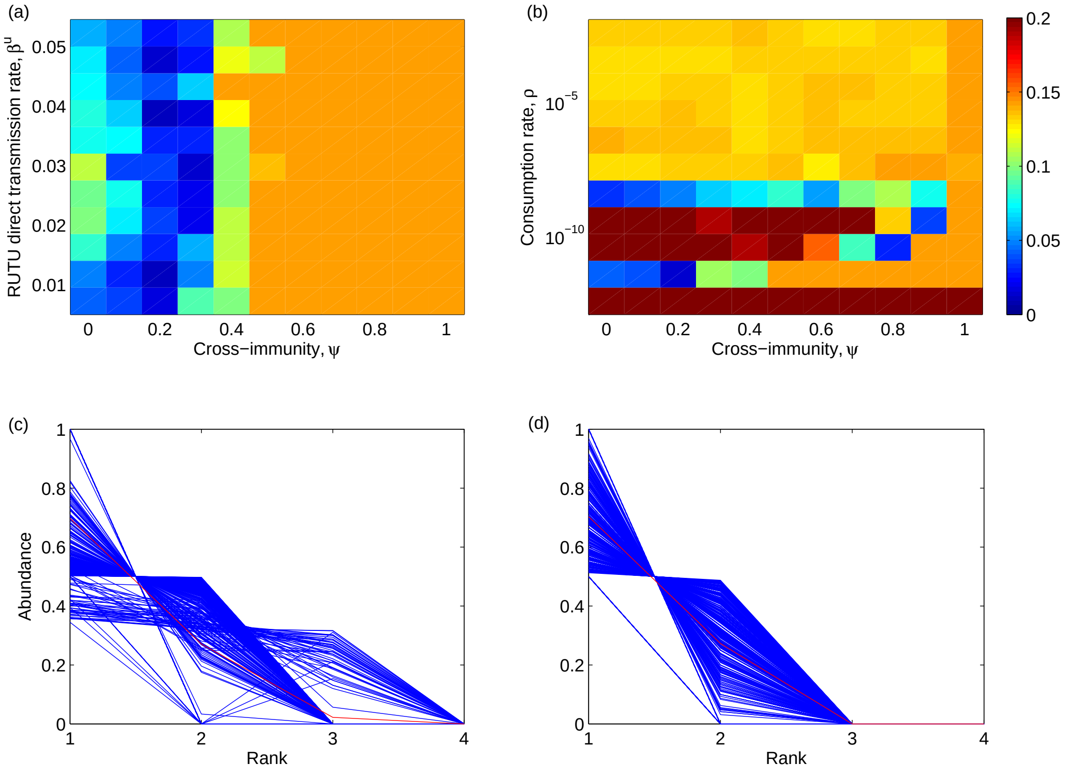

Figure S5. Panels (a) and (b) show the normalised SSEs for varying cross-immunity and either the ruddy turnstone transmission rate (a) or the consumption rate (b) against the metric rank-abundance. Panels (c) and (d) give the rank-abundance curves for the best fit SSE parameters for either (c) ruddy turnstone direct transmission rate β^u or (d) consumption rate ρ. These occur at a low level of cross-immunity ($\psi = 0.2$), with a low value for consumption rate (panels (b),(d); $\rho = 13804 * 10^{-16}$) or a high value for direct transmission rate (panels (a), (c); $\beta^u = 0.0365$).

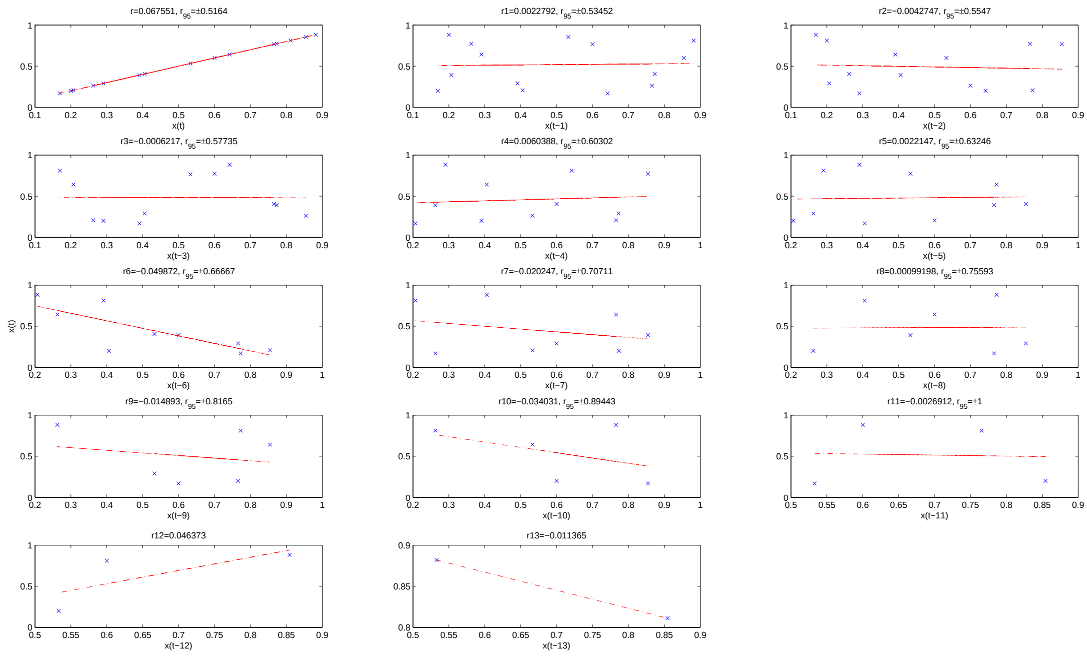

(a)

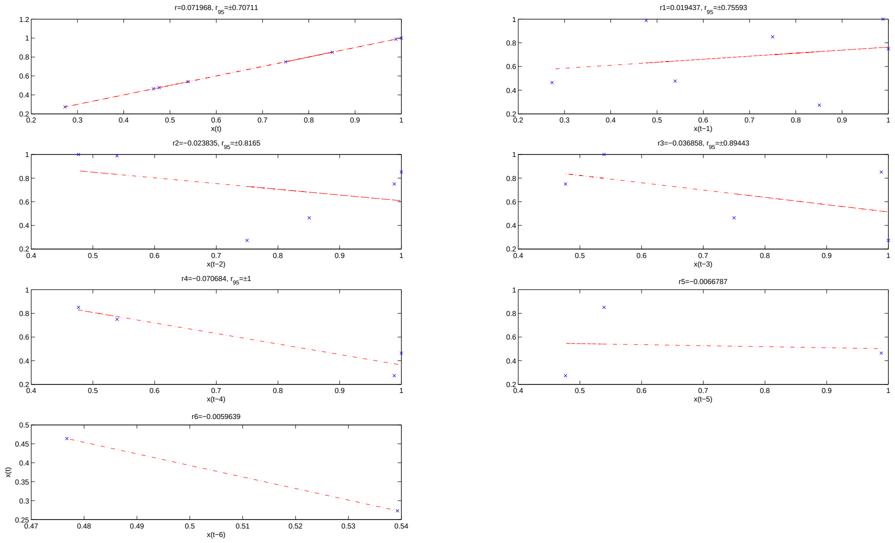

(b)

Figure S6. Lagged scatterplots showing for datasets 1 (a) and 2 (b), with linear fits also shown. Correlation coefficients are given above each plot and show none of the correlations are significant.

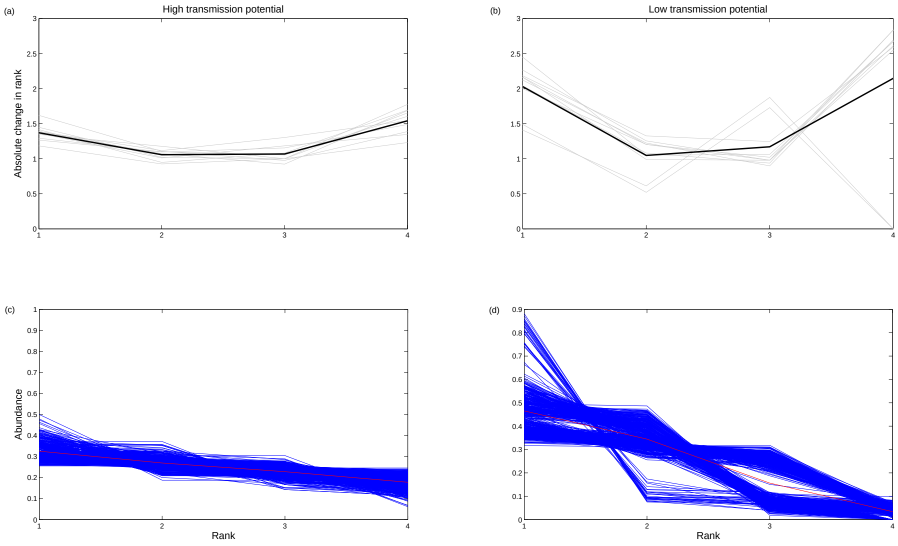

Figure S7. Change in rank ((a) and (b)) and rank abundance ((c) and (d)) plots for both high transmission potential ((a) and (c)) and low transmission potential ((b) and (d)).

a shape very similar to that seen in figure 4. Furthermore, the parameter sets modelled here represent a real challenge to the model - we varied multiple parameters at once, in all subtypes, that overall create a very different situation to that in the main text. That the model is capable of providing broadly similar results under these conditions supports its use in this work.

References

[1] Brown VL, Drake JM, Stallknecht DE, Brown JD, Pedersen K, et al. (2013) Dissecting a wildlife disease hotspot: the impact of multiple host species, environmental transmission and seasonality in migration, breeding and mortality. Journal of The Royal Society Interface 10.

[2] Burger J, Niles L, Clark K (1997) Importance of beach, mudflat and marsh habitats to migrant shorebirds on Delaware Bay. Biol Conserv 79: 283–292.

[3] Krementz DG, Conroy MJ, Hines JE, Percival HF (1988) The effects of hunting on survival rates of American black ducks. J Wildl Manage 52: 214–226.

[4] Anderson DR (1975) Population Ecology of the Mallard: V. Temporal and Geographic Estimates of Survival and Recovery and and Harvest rates., volume 125. United States Department of the Interior Fish and Wildlife Service Resource Publication.

[5] Delaware Department of Natural Resources and Environmental Control (2011). 2011-2012 Delaware Migratory Game Bird Season Summary. URL http://www.dnrec.delaware.gov/fw/Hunting/Documents/2011-2012%20Waterfowl%20Season%20Summary%20Sheet%20for%20website%2008122011%20final.pdf.

[6] Brown JD, Goekjian G, Poulson R, Valeika S, Stallknecht DE (2009) Avian influenza virus in water: Infectivity is dependent on pH and salinity and temperature. Vet Microbiol 136: 20–26.

[7] The Weather Channel L (2010). Monthly averages for Quebec, Canada. [WWW Document]. URL http://www.weather.com/outlook/travel/vacationplanner/vacationclimatology/monthly/CAXX0385. Accessed September 2010.

[8] The Weather Channel L (2010). Monthly averages for Lewes, DE. [WWW Document]. URL http://www.weather.com/weather/wxclimatology/monthly/USDE0030. Accessed September 2010.

[9] Climatetempinfo (2010). What is the climate, average temperature/ weather in Frobisher Bay, Baffin Island, Nunavut? when to go? [WWW Document]. URL http://www.climatetemp.info/canada/frobisher-bay-baffin-island-nunavut.html. Accessed October 2010.

[10] Nettleship DN (2000) Ruddy turnstone (*Arenaria interpres*). In: Poole A, editor, The Birds of North America Online, Ithaca: Cornell Lab of Ornithology; Retrieved from the Birds of North America Online: http://bna.birds.cornell.edu/bna/species/537. doi:10.2173/bna.537.

[11] BBC (2011). Average conditions, Belem, Brazil. [WWW Document]. URL http://www.bbc.co.uk/weather/world/city_guides/results.shtml?tt=TT001660. Accessed 2 Feb 2011.

[12] Schekkerman H, Slaterus R (2008) Population dynamics and prevalence of influenza A virus in mallard, mute swan and other wildfowl. Technical report, British Trust for Ornithology, Norfolk.

[13] Webster RG, Bean WJ, Gorman OT, Chambers TM, Kawaoka Y (1992) Evolution and ecology of influenza A viruses. Microbiol Rev 56: 152–179.

[14] Swayne DE, Slemons RD (2008) Using mean infectious dose of high- and low-pathogenicity avian influenza viruses originating from wild duck and poultry as one measure of infectivity and adaptation to poultry. Avian Dis 52: 455–460.

[15] Webster RG, Yakhno M, Hinshaw VS, Bean WJ, Murti KC (1978) Intestinal influenza: Replication and characterization of influenza viruses in ducks. Virology 84: 268–278.

[16] Francis CM, Sauer JR, Serie JR (1998) Effect of restrictive harvest regulations on survival and recovery rates of American black ducks. J Wildl Manage 62: 1544–1557.

[17] Metcalfe NB, Furness RW (1985) Survival, winter population stability and site fidelity in the turnstone arenaria interpres. Bird Study 32: 207–214.

[18] Latorre-Margalef N, Gunnarsson G, Munster VJ, Fouchier RAM, Osterhaus ADME, et al. (2009) Effects of influenza A virus infection on migrating mallard ducks. Proc R Soc B 276: 1029–1036.

[19] Maxted AM, Luttrell MP, Goekjian VH, Brown JD, Niles LJ, et al. (2012) Avian influenza virus infection dynamics in shorebird hosts. J Wildl Dis 48: 322–334.

[20] Gray J (2012) Worlds Out of Nothing, Springer London, chapter 13 - Across the Rhine Möbiuss Algebraic Version of Projective Geometry. pp. 149-159. doi:10.1007/978-0-85729-060-1_13.

[21] WHO (2012). Flunet. http://www.who.int/influenza/gisrs_laboratory/flunet/en/. URL \url{http://www.who.int/influenza/gisrs_laboratory/flunet/en/}.

Table S-1. Table of standard parameter values for each host species. The superscripts m, r, u stand for migrating ducks, resident ducks and ruddy turnstones respectively. These values are applied to all subtypes unless stated otherwise in the text.

Parameter	Symbol	Value/ Range	Unit	Source
Mallards				
Direct transmission (baseline)	β_0^m	0.01	year^{-1}	Parameterisation
Amplitude of seasonality	β_1^m	0.75		Parameterisation
Birth rate	b_0^m	2	year^{-1}	[12]
Average death rate	$\overline{\mu}^m$	0.5	year^{-1}	[12]
Recovery rate	γ^m	52	year^{-1}	[13]
Loss of immunity	ϵ^m	2.004	year^{-1}	Parameterisation
Consumption rate	ρ^m	$1.3804 * 10^{-12}$	year^{-1}	Parameterisation
Infection shape parameter	κ	100	EID$_{50}$	[14]
Shedding rate	ω^m	10^{12}	EID$_{50}$year^{-1}	[15]
Persistence	η	$4.9 - 42.6$	year^{-1}	[6]
American black ducks				
Direct transmission (baseline)	β_0^r	0.01	year^{-1}	Assumed $= \beta_0^m$
Amplitude of seasonality	β_1^r	0.75		Assumed $= \beta_1^m$
Birth rate	b_0^r	2	year^{-1}	[16]
Average death rate	$\overline{\mu}^r$	0.5	year^{-1}	[16]
Recovery rate	γ^r	52	year^{-1}	[13]
Loss of immunity	ϵ^r	2.004	year^{-1}	Assumed $= \epsilon^m$
Consumption rate	ρ^r	$1.3804 * 10^{-12}$	year^{-1}	Assumed $= \rho^m$
Infection shape parameter	κ	100	EID$_{50}$	[14]
Shedding rate	ω^r	10^{12}	EID$_{50}$year^{-1}	[15]
Persistence	η	$13.9 - 42.6$	year^{-1}	[6]
Ruddy turnstones				
Direct transmission (baseline)	β_0^u	0.005	year^{-1}	Assumed $= 0.5 * beta_0^m$
Amplitude of seasonality	β_1^u	0.5		Estimated
Birth rate	b_0^u	1.5	year^{-1}	[17]
Death rate	μ^u	0.15	year^{-1}	[17]
Recovery rate	γ^u	52	year^{-1}	[18]
Loss of immunity	ϵ^u	1	year^{-1}	[19]
Consumption rate	ρ^u	$1.3804 * 10^{-12}$	year^{-1}	Assumed $= \rho^m$
Infection shape parameter	κ	100	EID$_{50}$	-
Shedding rate	ω^u	10^{10}	EID$_{50}$year^{-1}	D. Stallknecht, *unpub.*
Persistence	η	$1.6 - 167.9$	year^{-1}	[6]
Predator shape parameter	θ	10^{-4}	year^{-1}	-
Number of horseshoe crabs	E	$1 - 10^5$		-
